# Communicatively Constructing the Bright and Dark Sides of Hope: Family Caregivers’ Experiences during End of Life Cancer Care

**DOI:** 10.3390/bs7020033

**Published:** 2017-05-15

**Authors:** Jody Koenig Kellas, Katherine M. Castle, Alexis Johnson, Marlene Z. Cohen

**Affiliations:** 1 Department of Communication Studies, University of Nebraska-Lincoln, Lincoln, NE 68588-0329, USA; kcastle4@unl.edu; 2 Department of Communication and Journalism, Arkansas Tech University, Russellville, AR 72801, USA; ajohnson93@atu.edu; 3 Center for Nursing Science, University of Nebraska Medical Center, Omaha, NE 68198, USA; mzcohen@unmc.edu

**Keywords:** hope, palliative care, cancer, communication, family caregiver, bereaved

## Abstract

(1) Background: The communication of hope is complicated, particularly for family caregivers in the context of cancer who struggle to maintain hope for themselves and their loved ones in the face of terminality. In order to understand these complexities, the current study examines the bright and dark sides of how hope is communicated across the cancer journey from the vantage point of bereaved family caregivers; (2) Methods: We analyzed interviews with bereaved family caregivers using qualitative thematic and case oriented strategies to identify patterns in the positive and negative lived experiences when communicating about hope at the end of life; (3) Results: Two overarching patterns of hope emerged. Those who experienced hope as particularized (focused on cure) cited communication about false hope, performing (faking it), and avoidance. Those who transitioned from particularized to generalized hope (hope for a good death) reported acceptance, the communication of hope as social support, prioritizing family, and balancing hope and honesty; (4) Conclusion: Family caregivers face myriad complexities in managing the bright and dark sides of hope. Interventions should encourage concurrent oncological and palliative care, increased perspective-taking among family members, and encourage the transition from particularized to generalized hope.

## 1. Introduction

The tension between hope for a cure and the reality of terminality [1] presents a communicative paradox for family caregivers in the context of cancer. Babrow, Kasch, and Ford [2] explain that illness is ripe with uncertainty. Family caregivers must help patients combat loneliness [3] and maintain their quality of life [4] as patients face their own terminality. Hope can influence what patients chose to disclose to family caregivers, and may hinder coping if the patient feels compelled to remain positive about their illness [4,5]. Thus, family caregivers play a vital role in helping patients move from hoping for recovery to hoping for a dignified death [6,7]. This endeavor is complicated, however, by family dynamics, as well as caregivers’ own needs, fears, burdens, and stressors. In short, a myriad of responsibilities may fall on the shoulders of family caregivers as they help patients and the larger family system negotiate hope at the end of life.

These responsibilities, as well as societal expectations, render hope particularly important for those facing serious illness [8], such as cancer. Indeed, hope plays a significant role in modern scripts for healthcare and the recovery process [8]. Hope is a vital component of what Frank [9] refers to as the restitution narrative, the dominant narrative of health and illness in which the body is viewed as starting in good health, descending into illness, and ultimately returning to good health. Illness is perceived as a bodily breakdown that physicians and patients must work to repair, positioning the physician as the active hero in the recovery process [9]. Hope, then, is an essential construct in maintaining the legitimacy and credibility of the medical model [10] situated within this master narrative.

Hope is also an important psychological resource at the end of life for cancer patients and caregivers. Hope can be a life-affirming [11] coping mechanism [12,13], giving meaning and protecting against despair [13]. Despite this, positioning hope as a welcome component of cancer care may be oversimplifying what is not only a psychological resource, but also a *communicative* construction, which must be negotiated by patients and caregivers who share in the cancer journey from differing vantage points.

Maintaining hope is complicated in the context of cancer, and patients and caregivers often have reported different experiences and needs. For example, Koenig Kellas, Castle, Johnson, and Cohen [14] found that whereas patients told cancer stories focused on themes of positivity in hope, and palliative and hospice providers worked to help families reframe cancer stories from curative to focused on everyday happiness, 40% of family caregivers in their sample experienced isolated cancer journeys marked by false hope and denial. The disproportionate number of family caregivers experiencing negativity and chaos in the cancer journey seems to support research on the stressors associated with caregiver burden [12] and made Koenig Kellas and colleagues reason that family caregivers may be particularly prone to experience isolation during cancer care. Indeed, while medical practitioners often postpone the severity of illness diagnoses in the context of cancer [15] and patients often avoid communication in the endeavor of protective buffering (i.e., protecting family members from psychological burden, [16]), family caregivers must increasingly bear the brunt of physical and emotional care. With this, family caregivers must broker the communication and negotiation of hope in an environment of shifting diagnoses, managing tension between patients and medical providers, keeping support networks and children appropriately apprised of the patients’ well-being, and providing needed levels of physical and psychological support.

Toward the end of life, when curative measures are no longer possible, family caregivers may experience tensions in shifting between hoping for a cure and accepting terminality. In the former, families hope that setbacks in patients’ illnesses are temporary and often focus on alleviating the symptoms that the patient faces [5]. As the disease progresses, family caregivers may move from a framework of particularized hope to one of generalized hope [17] shifting their focus from finding a cure to hoping for a good death [4,6] or a theologically/spiritually focused hope [15]. Generalized hope is a state of being that gives life meaning, while particularized hope is geared toward specific outcomes. Pattison and Lee [6] argue that, in the face of death, there is hope for a good afterlife. Family caregivers may turn to spiritual dedication or spiritual hope as a way of coping with illness and loss [18]. This transition can be difficult, however, and studies have shown that patients lament when their loved ones avoided discussing this transition and the realities of their illness, thereby hindering coping and well-being [19]. Denial can lead to false optimism [20] and to ill-advised treatment decisions [15].

Despite this, we know little about how hope is communicated in the face of cancer care, particularly in the experience of family caregivers. Understanding how family caregivers negotiate this tension is vitally important for their own and their family members’ well-being and end-of-life coping. Knowing the ways in which family caregivers experience and communicate hope during the cancer journey and at the end of life can lend insight into best and worst practices that may help others prepare for the immense responsibility of family caregiving and coping. Investigating these processes also acknowledges the potential functional ambivalence [21] of hope. In other words, hope is a construct typically regarded as positive, but, when considered in the complex context of cancer care and communicatively negotiated among the larger cancer caregiving system and family, can function with dispreferred outcomes. For example, family caregivers may be so focused on popularized notions that hope facilitates better patient outcomes (i.e., people who are hopeful beat cancer or live longer) that they may neglect the signs that patients are ready to move to generalized hope and prepare for death. Thus, in the current study, we were also interested in exploring the potentially bright and dark sides of hope as it is communicated in families in the context of cancer.

Through interviews with bereaved family caregivers, we investigated these positive and negative lived experiences with communicating about hope. Specifically, we posed the following research questions:

RQ1: How do family caregivers experience hope over the course of the cancer journey?
RQ2: How was hope communicated—by family caregivers and others—over the course of the cancer journey?

## 2. Materials and Methods

Participants were required to be at least 19 years of age and self-identify as a family caregiver for a patient in the context of cancer care. After receiving human subjects approval from the Institutional Review Board (IRB), we engaged in purposive sampling typical in health communication research [22] as well as snowball sampling [23]. First, flyers were distributed in palliative care clinics, local hospitals and oncology clinics, and at local community cancer events. Second, we posted the call for the study on social media (e.g., Facebook) and invited people to share the link to the recruitment materials.

The sample for the current study included 10 bereaved family caregivers who had provided primary or secondary care to their loved ones over the course of the cancer journey. Although this manuscript is part of a larger study in which we also recruited and interviewed cancer patients and medical practitioners, the current analysis focuses only on bereaved family caregivers so that we can best understand family communication of hope at the end of life. Interviews were conducted until theoretical saturation was reached on the two overarching themes described in the results. Participants included eight females and two males who ranged in age from 23 to 74 (*M* = 56.00, *SD* = 17.34). Participants were White and primarily middle class. They lived across a variety of Midwestern and Western states. Six of the participants were spouses of the cancer patients, one was a son, one was a daughter, one was a daughter-in-law, and one was a sister.

Interviews took place in person at a location of the participants’ choosing (e.g., the social interaction lab, coffee shops, office conference rooms, homes) or over the phone. They ranged in length from 18 to 115 min (*M* = 62.84, *SD* = 29.64) and resulted in 136 single spaced pages of transcription. After obtaining informed consent, one of the first three authors interviewed participants about their story of the cancer journey, questions about hope (e.g., “How has hope been present for you?” “Has anyone communicated hopeful messages to you over the course of the cancer journey and if so, what did they say, how did you feel, how did you respond?” “Has the communication of hope ever been unwelcome or negative?”), and questions about communication and relational challenges not relevant to the current analysis (see [7]). To ensure uniformity of the interviews, each of the first three authors met after our initial interviews to discuss the process and adjust the interview protocol and follow-up questions. The majority of changes were made to a portion of the interview protocol not relevant to the current study.

Interviews were transcribed verbatim and pseudonyms were provided to mask participant identity. We analyzed the data using a combination of qualitative case-oriented and variable-oriented strategies [24]. Specifically, we began by analyzing the data to answer RQ1 and RQ2 using thematic analysis guidelines offered by Braun and Clark [25]. We inductively identified themes, moving back and forth between the data and the emerging analysis. In order to get familiar with the data, we transcribed the interviews and then reviewed each transcription in depth. This process resulted in an initial list of categories relevant to the experience and communication of hope. We then coded the data and organized it into meaningful groups. This process yielded themes across cases for how bereaved family caregivers experienced hope (RQ1) and how they felt hope was communicated by and to them over the course of the cancer journey (RQ2). We then used a matrix approach to synthesize and compare individual cases in order to look for patterns in the data. This process of stacking comparable cases [24] (see Table 1) enabled us to examine the ways in which the interpretive recurring themes across cases justified creating two overarching types of hope experiences. These types of experiences—*Particularized* and *Particularized to Generalized*—were characterized by differing forms of communication of hope as presented in the Results below.

## 3. Results

The results of the thematic analysis revealed that participants experienced hope (RQ1) and communicated hope (RQ2) in both positive and negative ways. Indeed, the findings support the functionally ambivalent nature of hope in the context of cancer and end-of-life care. The results of the cross-case analysis revealed two overarching types of hope experiences that differed based on the experience of hope and the communication of hope across that experience. In what follows, we present the results of the thematic analyses organized according to these two emergent types of experiences: *Particularized* experiences of hope and hope that moved from *Particularized to Generalized.*

### 3.1. Particularized Hope

Research Question 1 asked about how bereaved family caregivers experienced hope over the course of the cancer journey. Consistent with previous literature on hope at the end of life [11], we found that bereaved family caregivers defined hope as either particularized—in which hope is geared toward a specific outcome (e.g., treatment, cure)—or as generalized—in which hope is geared toward some broadly defined future event (e.g., a good death, no pain). Particularized hope was illustrated by Ashley who described stages of hope that “kept not working out” (line 234). Ashley’s husband had pancreatic cancer and the family experienced a series of hopeful moments in treatment, including surgery followed by a clinical trial. She explains, “[There was hope] in the clinical trial. Um, there was hope that maybe that would work for him. And um it was hopeful for him, it was hopeful for me, it was hopeful for our kids. And, um, that made life a little easier to know that there was a grasp of hope” (lines 243–245). Whereas Ashley described stages of hope, Jackson described hope as a series of let downs. He described hope as particularized when he said, “Well everybody tries to give you hope and they tell you this might work. We have something new. And, you know, hope is a series of let downs. And nothing is a permanent cure, it just might get you a few more months, a year, who knows” (lines 46–48).

Some participants seemed in denial about the possibility of their loved one’s death from cancer, thereby keeping them in the stage of experiencing hope only as particularized. For example, Michelle never gave up on the idea of a cure for her mother’s lung cancer. Even after a surgery was cancelled due to complications, Michelle was unwilling to give up hope: “[The doctor said], ‘We’re not going to be able to do that procedure…we can’t do chemo...’ So, no chemo, no procedure. Nothing. They were just like we’re going to do what we can do to make her comfortable. So you know, I heard that, but I said, *you guys are going to fix her*” (lines 162–167, emphasis added).

Others seemed to want to move from a focus on hope as cure to hope for a good death (i.e., to move from particularized to generalized hope), but were unable to because of the unwillingness of other family members. For example, two wives in the sample—Karen and Sylvia—both discussed wanting to talk to their husbands about dying, but their husbands refused to give up hope that they would beat the cancer. Thus, although the family caregivers hoped to transition between stages of hope, their efforts were stymied by false hope and refusals from other family members, including patients.

Results of the cross-case data analysis (see Table 1) revealed that those who experienced hope as solely or primarily particularized also experienced the communication of hope similarly across the cancer journey. Specifically, in the current sample, 50% of the participants’ interviews and cancer stories were characterized only by the experience of particularized hope in which they hoped for a cure or the possibilities of treatment exclusively or until the very end of life. When they described how hope was communicated during their family’s cancer journey, their interviews were dominated by the communication of *false hope and minimizing*, *avoidance*/*denial*, and *performing (fake) hope*.

#### 3.1.1. The Communication of False Hope

Participants experienced the *communication of false hope* from medical practitioners, patients, and social network members. For example, Karen complained, “Oh practitioners always gave us hope I felt was very honestly…misleading, very misleading. I wish they would have been more up front about the progression of the disease…” (lines 171–173). Brian’s mother, during her battle with breast cancer tried to communicate hope to Brian: “My mom would always say, ‘It’s gonna be ok, I’m gonna be fine, you know this one’s gonna, this treatment’s gonna do it.’ And after the fifth or sixth treatment I realized that this wasn’t probably gonna work. So, I had no hope, pretty much” (lines 40–42). Finally, social network members communicated a type of false hope to participants when they minimized family caregivers’ expressed worry. Jackson explained, “Until there’s really a cure, you try to stay positive as you can, but you know, at first the people saying ‘You know everything’s going to be OK.’ The reassuring you helps, but then after a while it becomes white noise” (lines 116–118). False hope was experienced negatively by participants in the particularized group, but they were still not able to overcome it in the endeavor of moving to generalized hope.

#### 3.1.2. Performing Particularized Hope

Whereas others gave them false hope, family caregivers who remained primarily in a state of particularized hope also reported faking hope to others, something we coded as *Performing*. Performing entailed putting on a bright and positive face for others. Sometimes, this was embraced as part of a larger life attitude as in the case of Michelle who said, “I’ve always been hopeful of everything anyway, whether it’s been my mom’s situation or someone else’s. You know I’ve always been that person that says ‘Oh, everything will be fine. They are going to get through it because they are strong. It’s going to be OK’…with my mom, I fell off of that a little bit and blamed myself for her being ill and me not being able to fix things like I normally do. Since then, I’ve gotten back on track. And I’ve gotten back on hope lane and I’m back down that positive journey again” (lines 436–442). In turn, Michelle’s mother also performed for her. She said, “I would always tell my mom to stay positive and stay strong. As caregivers, we kind of have to be empathetic towards what they are going through… [but] I was selfish. Because I didn’t care that chemo was destroying her and making her miserable. I just wanted her to do it. And in the end she told my husband that she did it for me. Because she was tired. She told my husband that she was so tired. She said, ‘I’m doing this for Michelle’” (lines 403–408). Michelle’s mom performed for her long after she was ready to give up knowing it would keep Michelle’s hope alive.

Other participants put on a happy face for those around them as Jackson articulated, “Well, yeah, you know I tried to give hope to the actual patient, my wife. And tried to keep positive because what else can you do? Friends and family you also try to make less of it. You know you don’t want people hanging around thinking you know with long faces going, ‘Oh, I’m so sorry for you,’ um, you know, you want them upbeat. And you want them…happy around her that there is hope” (lines 62–66). Thus, particularized participants had to maintain (the illusion of) hope in order to maintain their particularized narrative of a cure.

#### 3.1.3. Avoidance/Denial

Finally, family caregivers who experienced hope as particularized reported the avoidance of communication about terminality or other difficult topics associated with end of life cancer. Sylvia, for example, lamented, “There were so many times I wanted to talk to him about how he felt about knowing he was going to die…but I just couldn’t because I knew he had this hope…and you know I never wanted to…discourage that because I thought, ‘you never know’” (lines 376–387). Karen also could not talk to her husband about death, but she also experienced avoidance from her extended family, including her husband’s brother who experience tremendous regret about his denial that his brother could die and the ensuing missed opportunity for communication. Moreover, her own parents refused to move from particularized to generalized hope in their communication. She notes, “My parents were one of these that didn’t talk about dying….I don’t know if it feared [sic] them that it might upset me, but I need it, I needed people to talk to. I needed people to understand what I was going through and what I feared” (lines 286–293). Karen’s needs were never met and her cancer journey was dominated by family avoidance and practitioner false hope.

#### 3.1.4. Summary

In short, half of the bereaved caregivers in the current study told stories and described hope in the context of end-of-life cancer as dominated by a particularized experience of hope. This experience was focused on cure and treatment. Therefore, in the endeavor of preserving hope, end-of-life family communication was dominated in these interviews by the communication of false hope (particularly by practitioners and social network members); the performance by family caregivers and other members to stay positive; and the avoidance of communication about death in the effort of preserving particularized hope.

### 3.2. Particularized to Generalized Hope

Unlike those whose journeys remained particularized, the other half of our sample told stories and described family end-of-life communication that was characterized by a shift between particularized and generalized hope. These participants hoped that treatment would cure their loved one’s cancer, but after realizing that the cancer was terminal, shifted their focus to hope for a good death, comfort, minimized pain, and/or an afterlife. The participants who experienced this shift too faced the communication of false hope and performing during the particularized phase of hope, but they also had interviews dominated by *acceptance* and the communication of hope in the forms of *social support* and the *prioritizing of family communication* as their experience shifted to generalized hope. Communication that was characterized as *performing* in those participants whose experiences stayed particularized, was softened by a shift to generalized hope for the other half of the sample. We coded this communication as the *Hope-Honesty Dialectic*.

#### 3.2.1. Acceptance

When participants shifted from particularized to generalized hope, they communicated a level of acceptance that was missing from the interviews of those whose hope remained particularized. Julia and her husband—who was diagnosed with esophageal cancer and lived for eight months—tried to be accepting of his prognosis from the very beginning, only experiencing the tension between particularized and generalized hope in their communication with extended family. Although she and her husband “struggled with family members over hope…presenting us with opportunities that in our mind offered false hope…We didn’t see it that way. We saw it as making the best of what time he had left” (lines 151–160). Julia communicated acceptance by supporting Josh’s decisions: “I remember the hope I gave Josh was that I would support the decision that he wants…I will not try to argue you out or into some kind of medical procedure…and be 100% with him in the decisions that we made together” (lines 217–226). Other caregivers held on to particularized hope longer, but eventually moved toward a generalized, more spiritual hope. When asked how hope was communicated in the family, Ashely said, “Well, I guess we just all talked about it and thought [treatment] would happen. And, uh, everybody was elated, but you know the stages kept not working out. So, I guess…everybody passes on eventually. And that this was just his time. And that nothing medically could prevent it. And we just kind of had to accept it. And not that it was easy, it wasn’t. We didn’t want to lose him. But, um, hope in that there is an afterlife” (lines 233–237). The move from particularized to generalized hope was enabled by an acceptance of terminality.

#### 3.2.2. The Communication of Hopeful Social Support

This kind of acceptance was facilitated in part by hopeful social support that family caregivers both gave and received. As one of her father-in-law’s caregivers, Sally communicated social support to her husband and mother-in-law as they negotiated the move from particularized to generalized hope. She remembered: “I don’t remember particular messages, but just reassuring them that it was okay for them to feel whatever they were feeling it was okay for them to be grieving, it was normal and they shouldn’t be feeling badly about feeling bad” (lines 219–222). Sally also received social support from her parents who she described as “huge in providing hope and encouragement for us [in that] they had just gone through this with my grandma…it was hopeful for us to know that…we weren’t abnormal in…what we were feeling…and also the hope that it was going to get better and easier” (lines 191–197). Lauren also described “a tremendous support system with my children… [and grandchildren]” telling the story of how their grandson would “go in and kneel by his grandfather’s bed and pray for him by the hour. Just holding his hand and wouldn’t let him go and telling him don’t give up. I think Jacob probably gave Ron the most hope of anyone, um, just because his faith was so strong…” (lines 373–376). Thus, when participants shifted from particularized to generalized hope, communication was characterized by hopeful messages of support.

#### 3.2.3. Prioritizing Open Family Communication at the End-of-Life

Participants whose hope journeys moved from particularized to generalized also had interviews dominated by intentional moves to prioritize open communication among family members and the connection it facilitated during cancer care. Diane traveled across several states to care for her brother at the end of his life and spent many hours talking to him, singing, and reminiscing about family. Ashley contrasted the difficulty in the transition from particularized hope to the hopeful communication that characterized generalized hope: “…Telling the kids that there was no more hope. It was difficult and it was difficult for them, of course. Um, but we were all together and… [crying] …our communication with each other was wonderful. I mean there wasn’t anything held back. We talked about everything and shared our opinions, and our advice, and our concerns” (lines 293–301). Julia, whose family resisted the move from particularized to generalized hope prioritized family communication when they finally came around: “The people that were the most helpful when his family finally came around and said, ‘OK we are embracing your plan and that means we’re going to come down and spend a weekend with you and we’re just going to have a really good time. And we’re going to talk about everything we want to talk about.’ And so those are the times that we really had a lot of hope that these were positive things that could come from his being sick and dying. You know, conversations he would have never had with people if he wasn’t dying that, you know, will never forget” (lines 200–206, emphasis added). At the transition of hope, open family communication was valued, prioritized, and facilitated closeness.

#### 3.2.4. Balancing Hope and Honesty

Finally, like those participants situated in the particularized experience of hope, those that moved from particularized to generalized hope also experienced the tension of wanting to *perform* positivity and hope for others. Their articulation emerged differently, however into what we coded as the balancing hope and honesty. This was the struggle between being situated with the acceptance of hope as generalized (i.e., being honest about the reality of death) as a family and maintaining a strong sense of positivity and hope, often for the dying loved one. Lauren stressed this in how she communicated the diagnosis with her children: “I think it’s very important to be as honest as you can be without saying that there’s no hope, but you do have to say this is a very serious situation. And, that prepares them, you know?” She also articulated this tension in how she evaluated patient–provider communication (“So there was always a glimmer of hope, but it was also I think very important for the doctor to be very honest and candid…there was always hope…prayer is powerful and faith will get you through. But reality has to be part of that picture of hope as well” lines 320–323). Finally, she directed family communication for her husband based on the hope–honesty dialectic: “I think that’s the one thing I did ask of all the children and grandchildren. When you visit with your grandfather don’t show him sadness if you can help it. Show him strength so that he’ll be able to let go and know that you’ll be OK” (lines 394–397). Sally also managed the hope–honesty dialectic in her communication with the larger support network. She explained, “I was the one sharing messages about ok, this is what happened at the doctor’s appointments, and I tried…to do it in a hopeful way. And I remember I would try to be positive no matter how bad the news was” (lines 214–217). Staying positive and hopeful even in the reality of facing death characterized the communication of those who transitioned in their journey of hope from cure to acceptance. It was less about performing and more about reframing and accepting death as inevitable and hopeful at the same time.

#### 3.2.5. Summary

In summary, one half of our participants moved from a particularized focus on treatment and cure to a hope that was generalized—focused on a good death, afterlife, and enjoying the time family members had left together. The experience of these participants was qualitatively different than the experience of those who remained in a particularized stage of hope as their communication was characterized by hope as social support, close family communication, and maintaining hope in the face of acceptance.

## 4. Discussion

The current analysis used a combination of thematic and case-oriented strategies [24] to identify two trajectories of hope for bereaved family caregivers. The two experiences—particularized and particularized to generalized hope—supports previous research that shows that many people move from hope for a cure to hope for a good death [6]. Our findings add to the literature in two important ways. First, our results provide an initial portrait of the communication that characterizes hope in families’ end-of-life cancer journeys. Participants who stayed in the particularized stage of hope experienced the communication of false hope, were required to perform, or “fake,” hope for others, and experienced avoidance and denial from family members in communication that may have allowed them to move from particularized to generalized experiences of hope. Those that did make the transition, on the other hand, experienced acceptance and hopeful communication in the forms of social support, prioritizing family communication, and negotiating the dialectic between honesty and communicating hope.

Although previous research examines the move between particularized and generalized hope, in hope theory, hope is conceptualized as a psychological construct independent of a relational context. McNulty and Fincham [26] (pp. 103–104) call for a more contextualized understanding of psychological constructs because “whether optimism or expectancies for desirable outcomes have beneficial or harmful implications depends on the context in which they occur”. This is particularly true when considering hope in the context of palliative care, and is further compounded when you consider the *communication* of hope given that communication constitutes lived experiences and relationships. After all, human existence is relational [27], and meaning is constructed between communicators [28]. People construct the meaning of hope together through communication, making the relational context of utmost importance in understanding hope. Whereas hope theory recognizes that interpersonal relationships are central to maintaining hope [29], it fails to recognize that hope itself is negotiated through interpersonal relationships wrought with tensions in understanding and experiencing hope. The current study lends texture to our understanding of hope as a communicated construct in families at the end of life.

Second, the current study illuminates the bright and dark sides of hope communication at the end-of-life. False hope has been characterized in previous research as problematic. When false hope is fostered by medical providers, for example, it can be especially damaging to the patient’s ability to adapt positively to their situation and importantly, to re-construct their identities in the face of their changing circumstances [13]. Additionally, it can influence their ability to be responsive to and engaged in their own care given that their goals and expectations of medical provider recommendations may be curative as opposed to palliative [30]. Previous research, however, focuses primarily on the effects of false hope on patients. The current study shows that family caregivers have much to balance in the effort of maintaining, not only medically-dictated but also family- and patient-dictated forms of false, particularized hope. Hope is a precarious balance for caregivers. Previous research reveals the beliefs that talking about cancer erodes hope thereby fueling silence [31]. This was seen in the avoidance strategies of particularized participants in our study. Strategies such as mutual topic avoidance and demand-withdraw patterns of conflict are inversely associated with marital satisfaction and predict psychosocial distress [32]. Thus, understanding the dark side of communicating hope and the inability to transition from particularized to generalized hope at the end of life has consequential outcomes for family health. In the current study, when the participants transitioned from particularized to generalized hope, they did seem more able to experience the brighter sides of hope communication—including deep connection with family members, affirming social support, and the ability to maintain positivity while still accepting the reality of their loved one’s terminality.

Despite the positive sides of hope, the amount of competing communication demands that family caregivers must manage in the practice of maintaining hope signals a paradox in the communication of hope at the end of life. Talking about moving from particularized to generalized hope (e.g., accepting the likelihood of terminality and facing death together) can erode hope associated with the restitution narrative [9]. Talking about moving from generalized to particularized hope (e.g., arguing for treatment by family members to patients or couples who have accepted terminality) may erode hope in narratives of acceptance. Not talking about the transition between types of hope precludes the potential hope offered by more biospychosocial approaches to supportive, family-based care—namely, palliative care.

Koenig Kellas and colleagues [14] argued for the need for concurrent oncological and palliative care and palliative family communication interventions focused on bridging the gaps between patients, health, and family caregivers. Although palliative care is intended for patients and their families at all stages of serious illness, the adoption of palliative care and biopsychosocial, patient-centered models of care still lag behind biomedical models [19]. This is unfortunate because cancer patients and their families experience reciprocal suffering that can be alleviated in palliative care [33]. Palliative care focuses on both curative and comfort care and on the spiritual, psychosocial, familial, and emotional needs of patients in addition to their physical needs [34] by taking a team-based approach to care—ideally from the time of diagnosis to death. The concurrent delivery of oncological and palliative care is in demand and supported by the American Society of Clinical Oncology’s Provisional Clinical Opinion for the combination of oncology and palliative care [35]. This sentiment is echoed by the Institute of Medicine (IOM)’s [36] recommendations for patient-centered care, developing caregiving competencies of the surrounding workforce, and the need for palliative care, psychosocial support, and family caregiver support across oncological care in its recent report, *Delivering High Quality Cancer Care: Charting a New Course for a System in Crisis*. The results of the current study suggest that some of those competencies may involve the negotiation of hope in a complex system of interpersonal and professional relationships. The path between particularized and generalized hope in the context of end-of-life palliative care is not always linear and family caregivers need guidance for their own and their families’ well-being. Palliative interventions are needed that encourage communication competence and the experience of cancer as communal (see [7]). In the current study, the communication of hope facilitated acceptance when generalized, through social support, and through the prioritization of family communication. These skills can be facilitated and improved among families in the context of cancer and other life-limiting illnesses.

Finally, the move from particularized to generalized hope seems to enable the possibility of effective, life affirming final conversations [37]. These conversations have been shown to benefit families, bringing closure and increasing closeness at the end of life. Family caregivers must help manage hope, but given its paradox, they must also receive training in conceptualizing, communicating, and reframing hope to enable family connection and individual well-being at the end of life. Future research should test the connections between managing hope and psychosocial health with larger samples than were possible in the collection of the current data.

## 5. Conclusions

Family caregivers face myriad complexities in managing the bright and dark sides of hope. Interventions should encourage concurrent oncological and palliative care, increase perspective-taking among family members, and encourage the transition from particularized to generalized hope.

## Figures and Tables

**Table 1:** Cross-Case analysis of hope experienced and the communication of hope.

Participant	Hope Experienced	Communication of Hope
Jackson	Particularized	Performing False Hope
Brian	Particularized	Performing False Hope
Sylvia	Particularized	Performing False Hope Avoidance/Denial
Karen	Particularized	False Hope Avoidance/Denial
Michelle	Particularized	Performing False Hope Avoidance/Denial
Julia	Particularized → Generalized	Acceptance Prioritizing Family
Ashley	Particularized → Generalized	Acceptance Prioritizing Family Social Support
Sally	Particularized → Generalized	Acceptance Social Support Balancing Hope & Honesty Performing
Lauren	Particularized → Generalized	Acceptance Social Support Balancing Hope & Honesty Performing
Diane	Particularized → Generalized	Social Support Prioritizing Family
